# Apparent *PT*-symmetric terahertz photoconductivity in the topological phase of Hg_1−*x*_Cd_*x*_Te-based structures

**DOI:** 10.1038/s41598-020-59280-0

**Published:** 2020-02-11

**Authors:** A. V. Galeeva, A. S. Kazakov, A. I. Artamkin, L. I. Ryabova, S. A. Dvoretsky, N. N. Mikhailov, M. I. Bannikov, S. N. Danilov, D. R. Khokhlov

**Affiliations:** 1 Physics Department, M.V. Lomonosov Moscow State University, Moscow, 119991 Russia; 2 Chemistry Department, M.V. Lomonosov Moscow State University, Moscow, 119991 Russia; 3 A.V. Rzhanov Institute of Semiconductors Physics, Syberian Branch of RAS, Novosibirsk, 630090 Russia; 4 P.N. Lebedev Physical Institute of RAS, Moscow, 119991 Russia; 5 Faculty of Physics, University of Regensburg, Regensburg, D-93053 Germany

**Keywords:** Topological insulators, Terahertz optics, Topological insulators, Terahertz optics

## Abstract

We show that the terahertz (THz) photoconductivity in the topological phase of Hg_1–*x*_Cd_*x*_Te-based structures exhibits the apparent *PT*- (parity-time) symmetry whereas the *P*-symmetry and the *T*-symmetry, separately, are not conserved. Moreover, it is demonstrated that the *P*- and *T*-symmetry breaking may not be related to any type of the sample anisotropy. This result contradicts the apparent symmetry arguments and means that there exists an external factor that interacts with the sample electronic system and breaks the symmetry. We show that deviations from the ideal experimental geometry may not be such a factor.

## Introduction

Recently, non-Hermitian physics and parity-time (*PT*)-symmetry became a matter of extensive experimental and theoretical studies^1–3^. The situations described by this formalism correspond to open systems, i.e. there exists an energy flow through the system under study. This concept has been successfully exploited in optics^4,5^, when an interplay between the optical gain and loss results, for instance, in an exciting example of a unidirectional invisibility of metamaterials^6,7^. In this paper, we demonstrate that features of the *PT*-symmetry may be realized not only in optical phenomena, but in photoelectric effects as well.

One of the interesting aspects in the physics of non-Hermitian systems is related to topological phenomena in optics^4,5^. This interest grew up from another hot topic in the solid state physics – studies of topological insulators^8,9^. In the 3-dimensional topological insulators, strong spin-orbit coupling results in a reversal of the energy terms corresponding to the conduction and valence bands in the semiconductor bulk. Consequently, topological 2-dimensional electron energy states necessarily appear at the surface of a 3D topological insulators. These states are characterized by the Dirac dispersion relation with the zero effective mass. Besides, the electron spin direction is locked perpendicularly to its momentum, thus preventing electrons from the backscattering. The two circumstances mentioned above make very attractive the idea to use the electron transport along the topological surface electron states in electronic devices.

Existence of the topological surface electron states was first confirmed experimentally for Bi_1−*x*_Sb_*x*_ and Bi_2_Se_3_ through the angle resolved photoemission spectroscopy^10,11^. Transport measurements in such 3D materials meet a substantial difficulty due to high number of electrically active growth defects. These defects give rise to a high free charge carrier concentration, so the bulk conductivity shunts the conductivity along the thin surface topological layer.

Another experimental approach used for instance in the papers^12–17^ implies an optoelectronic access to surface electron states. In many cases, photoelectric effects, such as photogalvanic effect^12–14^ and photoelectromagnetic effect^15–17^ are not sensitive to the bulk conductivity, even if the bulk free carrier concentration is high.

Solid solutions Hg_1−*x*_Cd_*x*_Te present a special case of the topological phase. First, the spin-orbit interaction decreases with increasing the Cd content *x* in the alloy. Therefore the electron energy spectrum is inverted and corresponds to the topological state at *x* < 0.16 at low temperatures, whereas it is direct at *x* > 0.16, so a trivial insulator phase is formed^18^. Consequently, it is possible to realize a transition between the topological and trivial phases by tuning the alloy composition. Note that in the topological phase at *x* < 0.16, the alloy is semimetallic, i.e. there is no energy gap between the light electron and heavy hole bands^19–21^. Moreover, modern techniques of the epitaxial growth such as the molecular beam epitaxy allow synthesis of Hg_1−*x*_Cd_*x*_Te films with low free carrier concentration ~10^14^ cm^−3^ ^22,23^.

In this paper, we show that the positive photoconductivity observed in the topological phase of Hg_1−*x*_Cd_*x*_Te is *PT*-symmetric since the simultaneous magnetic field reversal and change of the mirror-twin potential probe couple keeps the photoconductivity signal intact. At the same time, the photoconductivity signal is sensitive to the magnetic field direction and to the choice of mirror-twin potential probes across the sample thus breaking both the *T*- and *P*-symmetry, separately. In contrast to that, the THz photoconductivity in the trivial phase of Hg_1−*x*_Cd_*x*_Te is negative and conserves both the *P*- and *T*-symmetry.

## Experimental Results

We have studied 3.5 μm thick Hg_1−*x*_Cd_*x*_Te epitaxial films synthesized by MBE on top of a CdTe-rich mercury cadmium telluride buffer layer. Samples with the CdTe content *x* = 0.13, 0.15 and 0.17 were chosen for the experiments. The first two samples correspond to the topological phase with the inverted electron energy spectrum. The sample with *x* = 0.17 belongs to the trivial phase.

The Hall bar samples were placed into an optical helium cryostat, the sample temperature was kept at 4.2 K. Photoconductivity was induced by an optically pumped gas laser generating ~ 100 ns long Thz pulses with line tunable frequencies in the range from 0.6 to 3.3 THz^24–28^. The photoconductivity has been measured in the 4-probe geometry with two opposite directions of the *dc* bias current in order to avoid a possible contribution of the photovoltaic signal^29,30^. The propagation direction of the incident radiation and magnetic field were normal to the sample surface.

**Figure 1:**
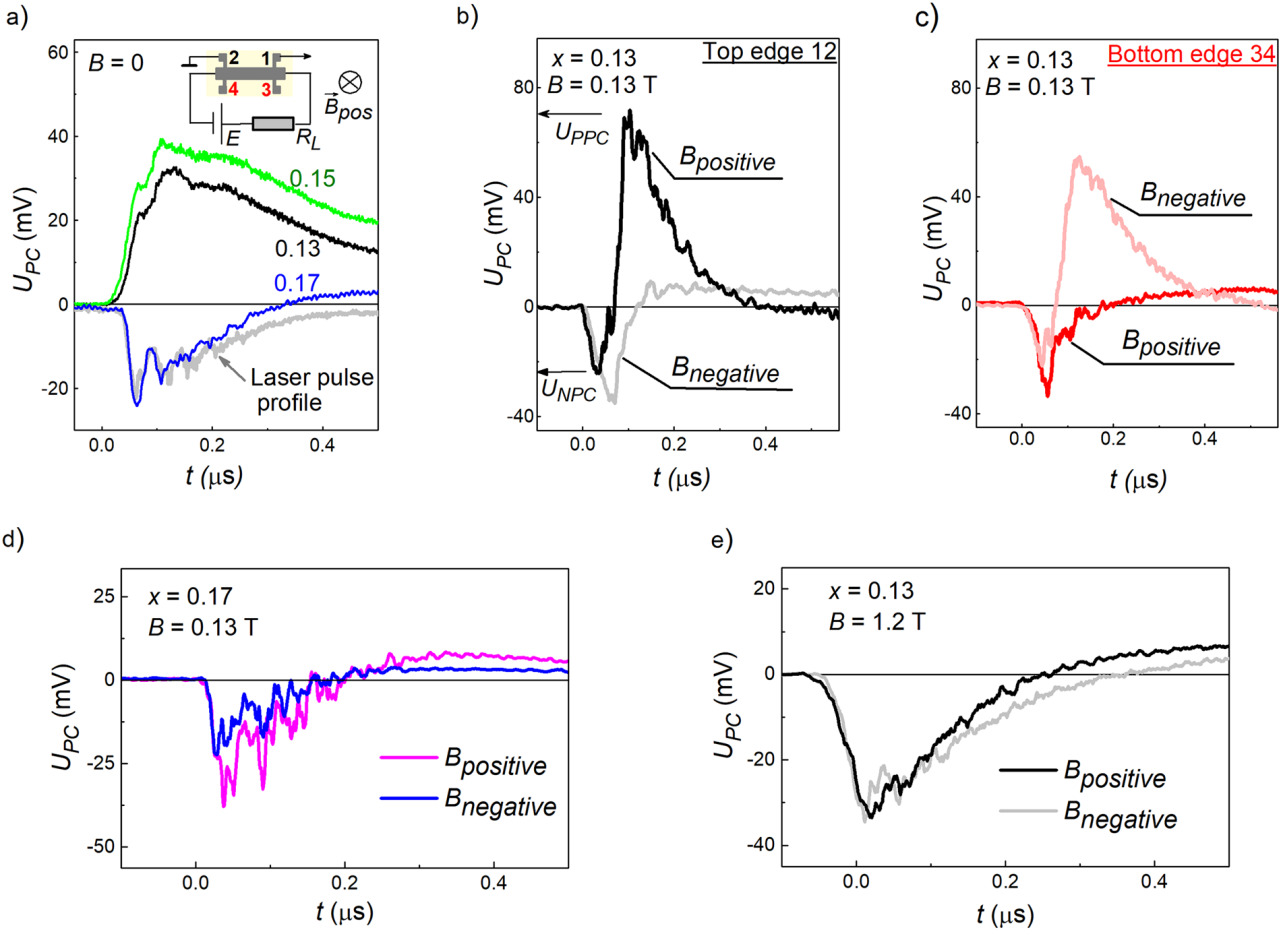
Kinetics of the photoconductivity signal *U*_*PC*_ excited by a THz radiation pulse. For the clarity, the positive *U*_*PC*_ values in the figure correspond to the positive photoconductive signal, the negative *U*_*PC*_ – to the negative photoconductivity. (**a**) Photoconductivity of films with different *x* (figures near the curves) obtained at the zero magnetic field. Signals are picked-up from the pair of contacts 1–2 made on the top edge of a Hall bar. The inset sketches the photoconductivity measurement setup. The grey curve shows the reference laser pulse shape over time in arbitrary units. (**b**–**e**) Photoconductivity signal measured along the top (**b**,**e**) and bottom (**c**) edges of a Hall bar sample with *x* = 0.13 (**b**,**c**) and *x* = 0.17 (**d**) subjected to the magnetic field *B* = ±0.13 T (**b**–**d**) and *B* = ±1.2 T (**e**). The positive magnetic field direction coincides with the incident radiation flux direction. We define the *U*_*PPC*_ and *U*_*NPC*_ values as amplitudes of the positive and negative photoconductive response, respectively.

Already at zero magnetic field the observed THz photoconductivity is qualitatively different for the topological and trivial phase samples. For the samples in the topological phase (*x* = 0.13 and 0.15), the photoresponse corresponds to the positive photoconductivity (sample resistance decreases upon irradiation) and is somewhat slower than the laser pulse, see green and black curves in Fig. 1(a). In contrast, for the trivial phase sample with *x* = 0.17 we observed fast negative photoconductivity, which follows the temporal shape of a laser pulse, see blue curve in Fig. 1(a). In all studied samples the overall behavior of the photoconductive response remains the same in the used radiation frequency range from 0.6–3.3 THz and is identical for contact probes along opposite edges.

Application of magnetic field qualitatively changes kinetics of the signal in the topological state samples, see Fig. 1(b,c), whereas for the trivial phase sample it remains the same, see Fig. 1(d). Now in samples with *x* = 0.13 and 0.15, kinetics of the photoconductive signal depends on the magnetic field strength and even direction. Moreover, it becomes different for signals picked-up from the contacts along the top and bottom edges of Hall bars.

**Figure 2:**
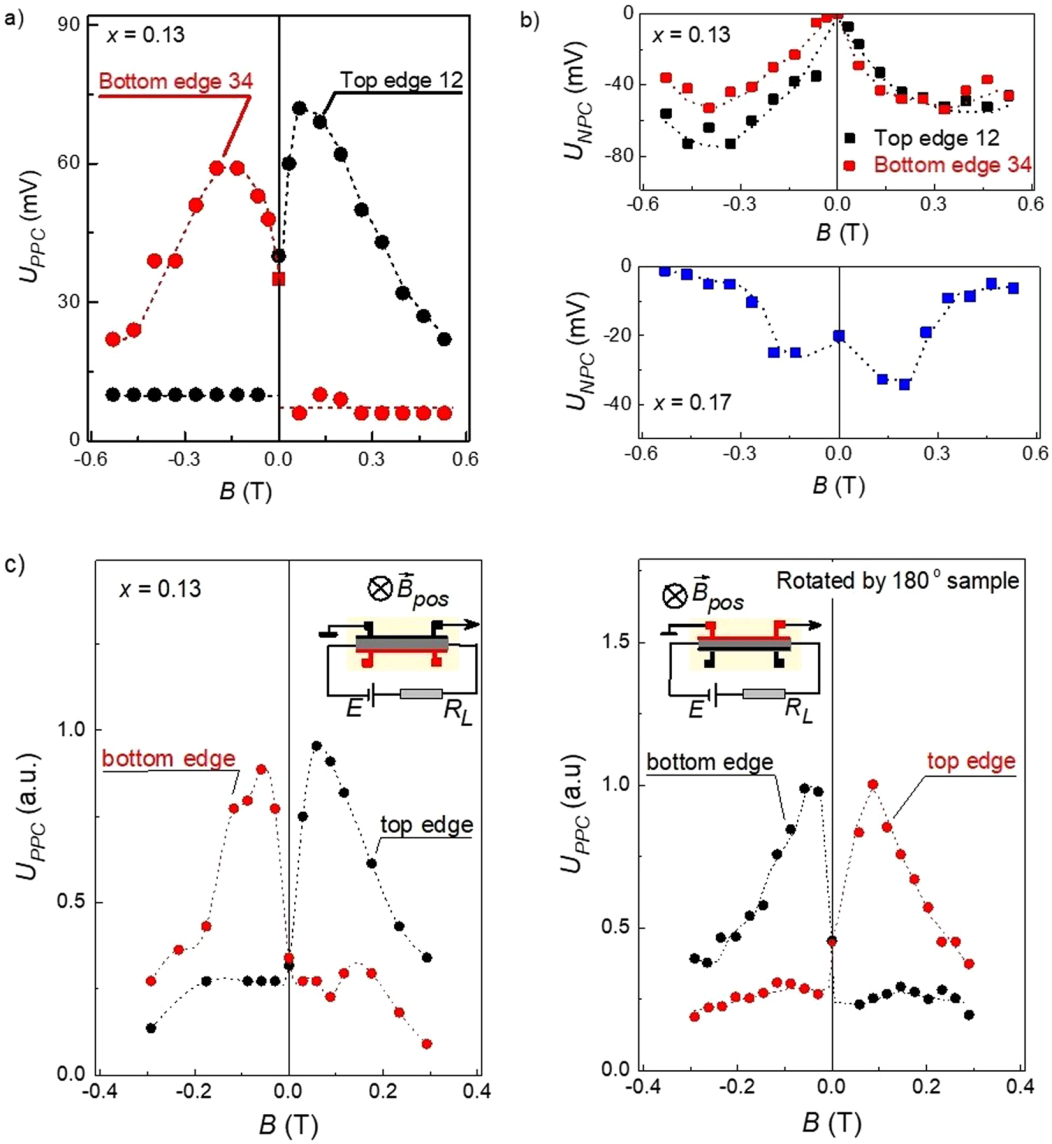
Magnetic field dependence of the photoconductivity amplitude. (**a**) Positive photoconductivity response amplitude *U*_PPC_ measured for the top (contacts 1–2) and bottom (contacts 3–4) edges of the topological phase sample with *x* = 0.13. (**b**) Negative photoconductivity amplitude *U*_NPC_ obtained for the top and bottom edges of the sample with *x* = 0.13 (top panel) and the topologically trivial sample with *x* = 0.17 (bottom panel). The THz radiation frequency *f* = 1.07 THz, the sample temperature *T* = 4.2 K. (**c**) Positive photoconductivity response amplitude *U*_PPC_ normalized to its maximal value, taken before (left panel) and after (right panel) rotation by 180 degrees around the magnetic field direction. The top and bottom edges of the sample are physically swapped under this operation. The physical edges are shown by the color, their positions (top or bottom) are indicated near each curve. The sample is in the topological phase (*x* = 0.13). The insets show the electric measurement circuit and the position of the respective sample edges.

Let us first describe the results for the top edge of a Hall bar. For positive magnetic field direction coinciding with the incident radiation flux *B*^+^, we observed dynamical change of sign of the photoconductive signal, see the black curve in Fig. 1(b). For *B*^+^ > 1 T, the slow positive photoconductivity disappears and the signal becomes dominated by the fast negative one, see Fig. 1(e). For negative magnetic fields, in contrast, the zero *B*-field positive photoconductivity almost vanishes in small fields *B*^*−*^ ≤ 0.13 T, and we detect only fast negative photoconductivity signal, see grey curve in Fig. 1(b). Strongly asymmetric magnetic field dependence of the positive photoconductivity amplitude *U*_PPC_ is shown in Fig. 2(a).

The fast negative photoconductivity, in contrast, is symmetric with respect to the magnetic field direction, see Fig. 2(b), top panel. Similar behavior of the negative photoconductive response amplitude is detected for the trivial phase sample with *x* = 0.17, see bottom panel of Fig. 2(b).

Strikingly, the described above behavior mirrors for the opposite edge of a Hall bar, see Figs. 1(c) and 2(a). Indeed, in contrast to the upper edge, at the lower edge, the sign-alternating photoconductive signal is detected for the negative magnetic field direction, whereas for positive magnetic fields, the photoconductivity becomes negative even for small magnetic fields, see respectively rose and red curves in Fig. 1(c). A comparison of magnetic field dependences of the positive photoconductive signal amplitudes obtained for the opposite edges shows that they are just reflected with respect to the zero magnetic field, see Fig. 2(a). The magnetic field dependence of the negative photoconductivity, however, is very analogous for both edges (see Fig. 2(b), upper panel).

Surprisingly, rotation of a sample by 180 degrees in the plane normal to the magnetic field direction does not affect the effects observed: for the positive magnetic field direction the positive photoconductivity is still observed at the top sample edge, whereas the negative photoconductivity remains at the bottom potential probe couple (Fig. 2(c)). All other features of the photoconductivity also remain intact. Note that physically, the operation of 180 degree rotation swaps the upper and lower sample edges.

The effects described above do not depend on the radiation polarization, neither circular, nor linear.

## Discussion

Application of an external magnetic field strongly affects both kinetics of the photoresponse and its magnitude. At a first glance, the observed asymmetric magnetic field dependence of the photoconductivity, being mirrored for opposite edges, could be explained by the Hall effect. Indeed, for a fixed direction of the bias current, the Lorentz force should push carriers to one of the edges for one magnetic field polarity, and to the other for the opposite one. This, however, contradicts with the observation that the photovoltage asymmetry does not change by switching direction of the bias current, see Fig. 2 in the Methods section. The figure shows that the reverse of the bias current direction only changes the signal polarity, which indicates that it is caused by photoconductivity, but it does not change its magnitude. The latter must be present for the Hall effect. Furthermore, the negative photoconductivity in both topologically trivial and nontrivial materials shows an almost symmetric dependence on magnetic field, see Fig. 2(b), being again against the Hall effect model. Thus the Hall effect either in the bulk or in surface state layers can be ruled out as a possible source of the asymmetric magnetic field dependence of the positive photoconductivity.

In fact, reversal of the magnetic field direction is equivalent to the time reversal; consequently, the positive photoconductivity is not *T*-symmetric. It is not *P*-symmetric as well since this symmetry is broken for mirror-like edges of the samples. At the same time, simultaneous change of the sample edge and the magnetic field direction keeps the photoconductivity intact, so it is *PT*-symmetric.

We call the effect observed “the apparent *PT*-symmetric photoconductivity” since it possesses apparent features of the *PT*-symmetric phenomena observed in optics, i.e. the *P*- and *T*-symmetries, separately, are broken, but the *PT*-symmetry is conserved. Beside that, the effect is observed only upon the THz excitation meaning that there exists an energy flow over the system under study. On the other hand, for the *PT*-symmetric effects in optics, a clear physical mechanism was suggested, which is not the case for the present study.

In general, breaking of the photoconductivity *P*- and *T*-symmetries contradicts the apparent symmetry arguments since it is not clear how does the system choose the sample side at which the positive photoconductivity is observed for a given magnetic field direction. The most logical reason for this asymmetry could be related to some sort of an in-plane anisotropy of surface topological layer features. However, the asymmetric positive photoconductivity is not affected upon rotation of a sample by 180 degrees in the plane normal to the magnetic field direction. If the *P*- and *T*-symmetry breaking would result from sample anisotropy, then, for a given magnetic field direction, the enhanced positive photoconductivity would be linked to a certain physical side of a sample, and it would rotate together with the sample. Experimentally, the opposite picture is observed. This fact rules out any sort of anisotropy that is linked to the sample under study. The only remaining option is the action of a factor that is external with respect to the sample.

We have studied factors related to a possible deviation of the experiment geometry from the ideal one: existence of non-normal components of the magnetic field, non-normal radiation incidence angle, non-homogeneous laser intensity distribution along and across a sample. It was shown that the *PT*-symmetric photoconductivity is very stable with respect to these parameters (see Supplementary materials). It means that there exists another unaccounted external factor that breaks the in-plane symmetry.

Clearly more experimental work is needed to extract a possible contribution of the structure elements including the film bulk and interfaces between the buffer, the film and the cap layer. On the other hand, appearance of the effective *PT*‐symmetric behavior of such a structure is an experimental fact that is repeated for many samples. Further experimental and theoretical studies are needed to probe the origin and scope of these parity-time symmetry phenomena in topological phases.

## Summary

In summary, we have demonstrated that the positive photoconductivity induced by THz laser pulses in heterostructures based on thick topological phase Hg_1−x_Cd_x_Te films has *PT*‐symmetrical features in magnetic field. We have shown that the appearance of this *PT*-symmetric photoconductivity cannot be associated with any sort of sample anisotropy. This result contradicts apparent symmetry arguments. It means that there exists an unaccounted external factor that breaks the system symmetry.

## Methods

### Sample synthesis and preparation

**Figure 3:**
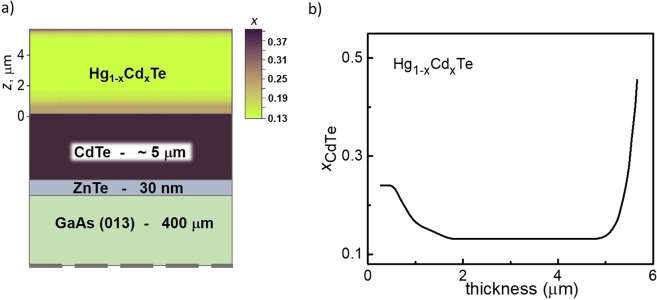
Samples under study. (**a**) A schematic presentation of the layer sequence in the heterostructures. (**b**) A typical distribution of the Hg_1−x_Cd_x_Te alloy composition throughout the structure thickness.

The Hg_1−x_Cd_x_Te epitaxial films studied were grown by molecular beam epitaxy. Single crystalline semi-insulating GaAs with the (013) orientation was used as a substrate. First, a 30 nm ZnTe buffer was grown to maintain the growth orientation. It was followed by a relaxed 5.5 μm thick CdTe layer. The epitaxial film studied was sandwiched between the graded gap Hg_1−x_Cd_x_Te 1.5 μm thick layer and a cap Hg_1−x_Cd_x_Te layer with the thickness of 0.5 μm. A schematic presentation of the layer sequence in the heterostructures studied is presented in the Fig. 3a. A composition and thickness of all elements of the heterostructure was controlled by ellipsometry. Fig. 3b shows the typical composition distribution throughout the thickness. The growth technique is described in detail in the papers^22,23^. Hall bars with the lateral dimensions of 6 × 0.5 mm^2^ were prepared by the standard photolithography (see insert in the Fig. 2). The distance between the potential probes was 2 mm.

### Sample characterization

Equilibrium galvanomagnetic characteristics of the samples were measured at 4.2 K. All samples possessed the *n*-type conductivity. The free electron concentration varied in the range (3.7–5.2)·10^14^ cm^−3^ for different samples, the electron mobility was in the range 5.8·10^5^–1.2·10^6^ cm^2^/V·s. These values were calculated in the assumption that the electron density is homogeneously distributed throughout the film. The energy distance between the Fermi level and the conduction band edge varied from 2 to 5 meV for different samples^29,30^. The latter value is less than the energy of a radiation quantum with the wavelengths of 280 and 496 μm.

### Experimental

The samples were mounted into an optical helium cryostat, the sample temperature was kept at 4.2 K. Photoconductivity was induced by an optically pumped gas laser generating ~ 100 ns long THz pulses with the power up to 7 kW at the wavelengths of 90, 148, 280 and 496 μm. The incident radiation power was monitored by a fast photon drag reference detector. The beam had an almost Gaussian profile, which was measured by a pyroelectric camera. The incident radiation was normal to the sample surface. Magnetic field up to 4 T directed perpendicular to the sample surface could be applied.

**Figure 4:**
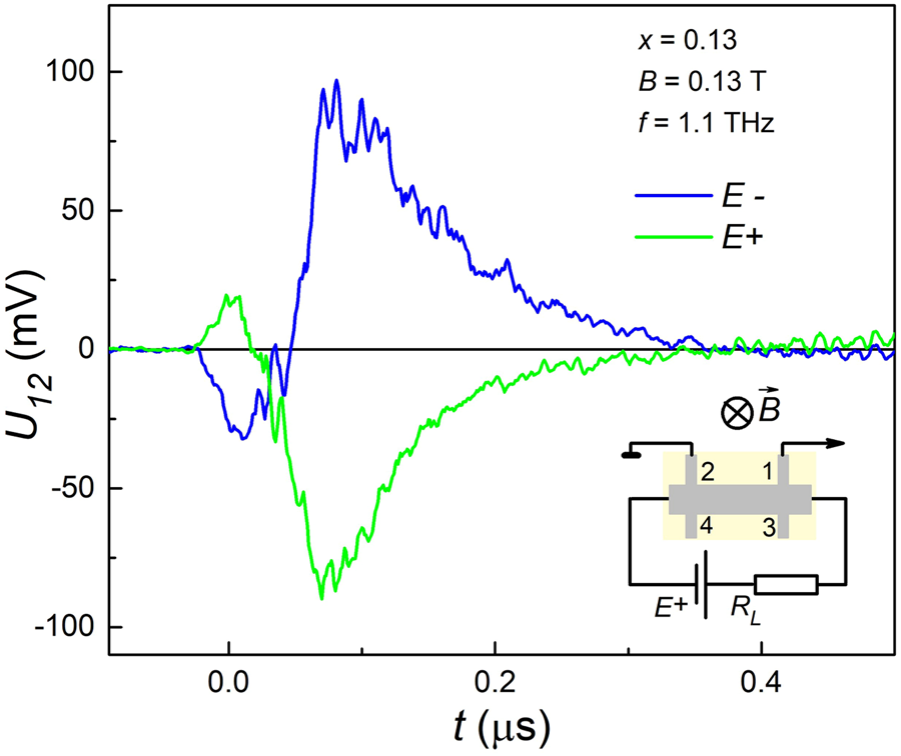
Photoconductivity kinetics for the opposite signs of external bias applied. Typical kinetics of the photoinduced voltage drop between potential leads 1–2 for two opposite bias polarities. The insert shows schematically the experiment geometry.

The photoconductivity has been measured in the 4-probe geometry with two opposite directions of the current to avoid possible contribution of the photovoltaic signal. Fig. 4 shows a typical kinetics of the photoinduced voltage drop between potential leads for two opposite battery polarities. It can be seen that the effect observed corresponds to the photoconductivity, not to the photovoltaic response, since its sign changes to the opposite one with the current reversal.

**Figure 5:**
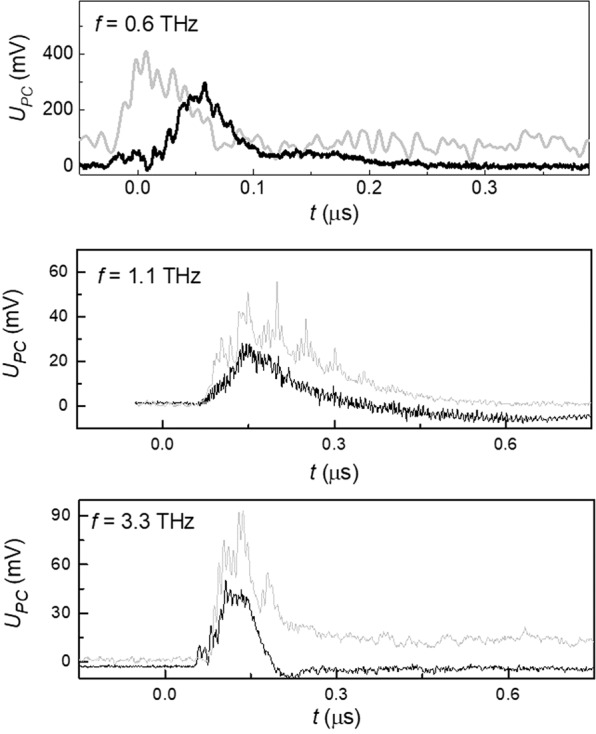
Photoconductivity kinetics for different incident radiation wavelengths. Kinetics of the positive photoconductive response taken for the topological phase sample with *x* = 0.15 for three different laser wavelengths in the zero magnetic field at *T* = 4.2.

The key features of the photoconductivity kinetics did not depend on the laser wavelength. As an example, the temporal shape of the photoconductivity taken in the zero magnetic field is shown for the sample with *x* = 0.15 in the Fig. 5.

## Supplementary information

Supplementary information. 

## Data Availability

Data that support the plots within this paper and other findings of this study are available from the corresponding author upon reasonable request.
