# Supplementary material for: Apparent PT-symmetric terahertz photoconductivity in the topological phase of Hg1−xCdxTe-based structures
Source: Sci Rep. 2020 Feb 11;10:2377. doi: 10.1038/s41598-020-59280-0 (PMC7012871; doi:10.1038/s41598-020-59280-0)
Supplement: Supplementary file 1 — Supplementary information. [file 41598_2020_59280_MOESM1_ESM.pdf]

Apparent *PT*-symmetric terahertz photoconductivity in the topological phase of $Hg_{1-x}Cd_xTe$-based structures

A.V. Galeeva[1], A.S. Kazakov[1], A.I. Artamkin[1], L.I. Ryabova[2], S.A. Dvoretsky[3], N.N. Mikhailov[3], M.I. Bannikov[4], S.N. Danilov[5], D.R. Khokhlov[1,4,*]

[1]Physics Department, M.V. Lomonosov Moscow State University, Moscow 119991, Russia
[2]Chemistry Department, M.V. Lomonosov Moscow State University, Moscow 119991, Russia
[3]A.V. Rzhanov Institute of Semiconductors Physics, Syberian Branch of RAS, Novosibirsk 630090, Russia
[4]P.N. Lebedev Physical Institute of RAS, Moscow 119991, Russia
[5]Faculty of Physics, University of Regensburg, Regensburg D-93053, Germany
*khokhlov@mig.phys.msu.ru

Supplementary information

Stability with respect to deviations from the ideal geometry

The ideal Faraday geometry presumes the magnetic field direction normal to the sample surface and normal radiation incidence angle. We have studied the effect of possible deviations of the experiment geometry from the ideal one.

First, we have checked how does the zero-field positive photoconductivity amplitude depend on the radiation incidence angle α. The α value varied from -60º to +60º with respect to the nominal normal incidence angle in the plane parallel to the long side of the Hall bar (see inset in the Fig.1S). The results are shown in the Fig.1S, black points. It can be clearly seen that the positive photoconductivity amplitude practically does not depend on the radiation incidence angle. This result is understandable if we take into account that the radiation intensity used in our experiment corresponded to saturation of the photoconductivity, so even much weaker radiation has caused practically the same photoresponse.

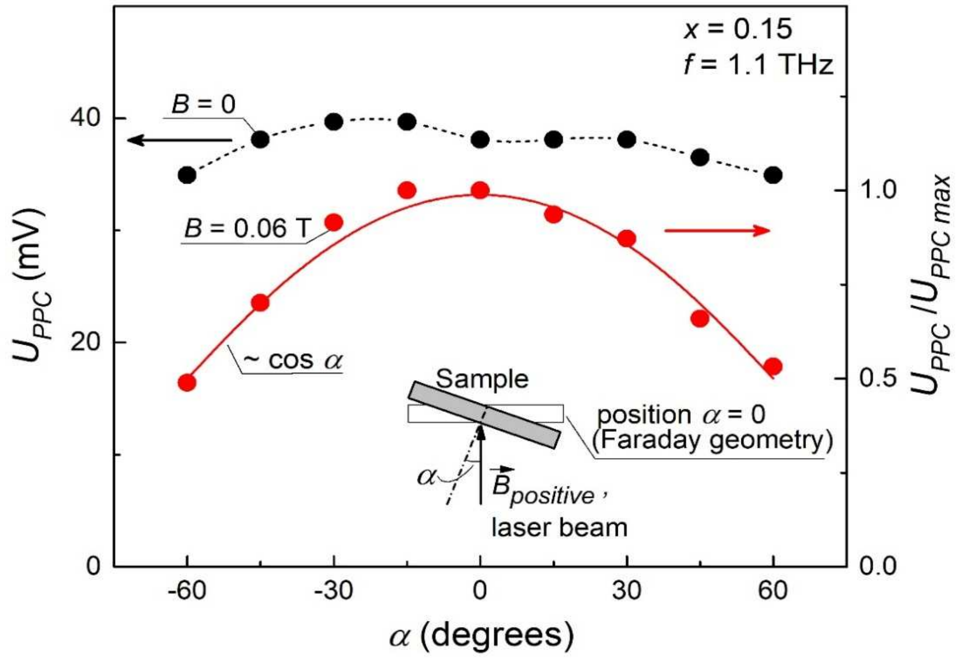

Supplementary Figure 1S | Angle dependence of the photoconductivity amplitude. Black points: dependence of the positive photoconductive response amplitude U_{PPC} on the radiation incidence angle α in the zero magnetic field. Red points: dependence of the relative positive

photoconductive response amplitude U_{PPC}/U_{PPCmax} on α in magnetic field $B = 0.06$ T. The inset shows the experiment geometry.

The angle dependence of the positive photoconductivity amplitude taken in magnetic field corresponding to the maximal positive photoconductivity signal $B = 0.06$ T, is shown in the Fig.1S, red points. It follows well the $U_{ppc} \sim \cos\alpha$ dependence suggesting that the normal component of the magnetic field is responsible for the effect. In any case, in a wide α range from -30º to +30º the photoconductivity amplitude varied by no more than 10%. This result means that a small non-normal magnetic field component directed along the Hall bar does not affect the photoconductivity amplitude.

Let us turn now to a possible in-plane magnetic field component directed across the Hall bar. To account for that, we have performed the following experiment. The sample was rotated by 180º *in situ* around the vertical axis pointing across the Hall bar in the sample plane (see insets in the Fig.2S), and the magnetic field polarity was swapped. The new configuration was also of the Faraday type with two distinctions from the previous case. The first one is that the sample was illuminated from the substrate. The second distinction is the following. If there would exist an in-plane magnetic field component across the Hall bar in the initial configuration, it would change its direction to the opposite one in the new configuration. So if this magnetic field component would be a factor breaking the system symmetry, the photoconductivity would change its sign upon such a manipulation. The experimental results are shown in the Fig.2S It can be clearly seen that this geometry transformation practically does not affect the photoconductivity suggesting that the effect is robust with respect to small possible deviations of the magnetic field direction from the ideal Faraday geometry.

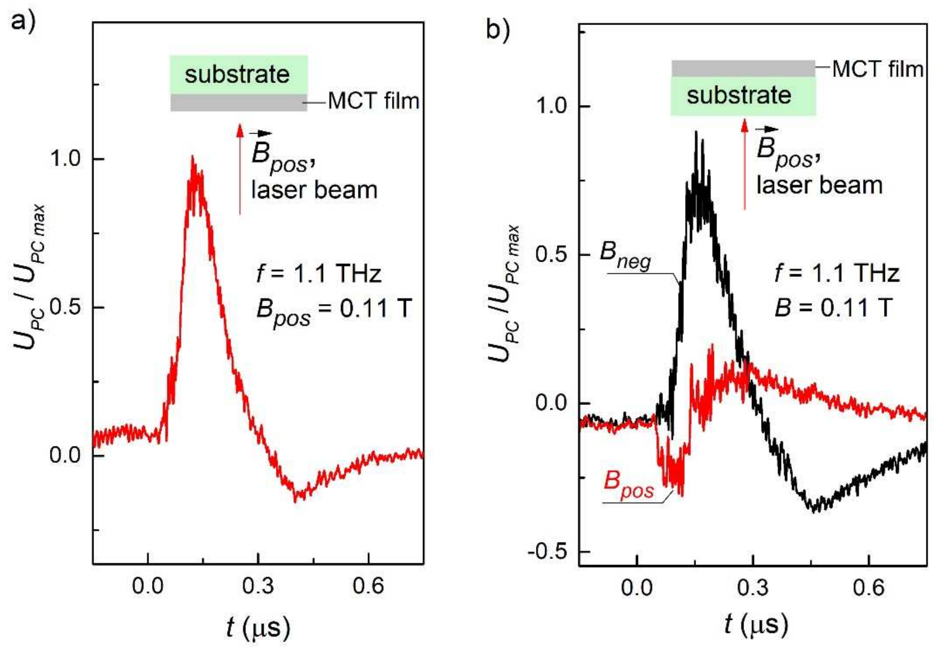

Supplementary Figure 2S | Kinetics of the photoconductivity with opposite directions of incident radiation. Kinetics of the photoconductive response normalized to its maximal value before and after rotation of a sample with $x = 0.15$ by 180º around the vertical axis. (a) Photoconductivity kinetics for the illumination from the film side. The positive magnetic field value is 0.11 T. (b) Photoconductivity kinetics for the illumination from the substrate side for the same magnetic field direction as in the panel (a) (red curve), and for the opposite magnetic field direction (black curve).

To study a possible influence of the incident radiation intensity gradient, the laser spot was moved along and across the Hall bar. The range of the laser spot motion is shown in the inset to the Fig.3S (a). The positive photoconductivity amplitude was measured at $B =$ 0.11 T for the opposite magnetic field directions. The respective typical photoconductivity kinetics is presented in the Fig.3S(a). The Fig.3S(b), top panel, shows dependence of the positive photoconductivity amplitude U_{PPC} for the two magnetic field directions in three laser spot positions: in the Hall bar center, and at right and left Hall bar sides. The Fig.3S(b), bottom panel demonstrates analogous data for the laser spot located in the Hall bar center, and at its upper and lower edges. It is clearly seen that the initial asymmetry of the photoconductivity in magnetic field remains irrespectively on the exact position of the laser spot on a sample. Note that the laser beam has an almost Gaussian profile with the characteristic size varying from 1 mm to 3 mm depending on the wavelength. As it was mentioned earlier, the positive photoconductivity amplitude saturates for the radiation intensity power used in our experiments. It means that even the "wings" of a laser spot with smaller intensity provide practically the same photoconductive response.

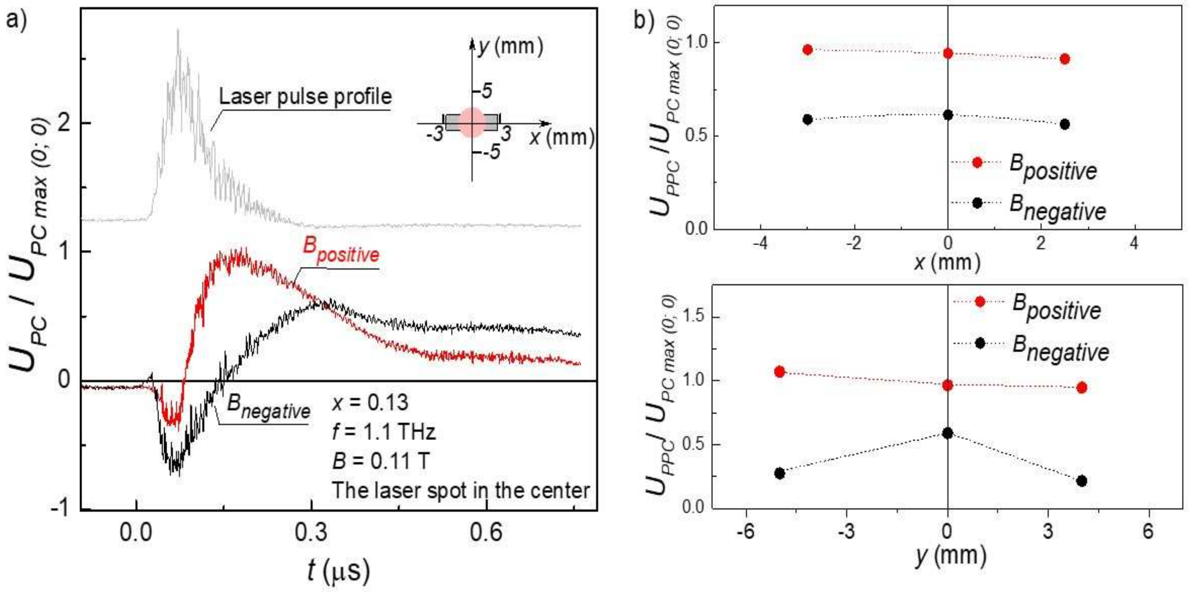

Supplementary Figure 3S | The photoconductivity dependence on the laser spot position. (a) Kinetics of the photoconductive response normalized to its maximal amplitude taken for two opposite magnetic field polarities for the laser spot center located in the Hall bar center. The inset shows the actual Hall bar size and the range of the laser spot motion. The magnetic field amplitude is 0.11 T, the radiation frequency is 1.1 Thz, the $Hg_{1-x}Cd_xTe$ film composition $x = 0.13$. (b) Dependence of the normalized maximal positive photoconductive response on the laser spot position along (top panel) and across (bottom panel) the Hall bar.
